# Genetic Variants of BMI1 (rs1042059 and rs11591377) and Their Potential Role in Leukemia Susceptibility: A Narrative Review

**DOI:** 10.1002/hsr2.72579

**Published:** 2026-06-19

**Authors:** Mojtaba Aghaei, Niloufar Afrough, Mohammad Ali Jalali Far

**Affiliations:** ^1^ Thalassemia & Hemoglobinopathy Research Center, Health Research Institute Ahvaz Jundishapur University of Medical Sciences Ahvaz Iran; ^2^ Student Research Committee Ahvaz Jundishapur University of Medical Sciences Ahvaz Iran; ^3^ Department of Laboratory Sciences, School of Allied Medical Sciences Ahvaz Jundishapur University of Medical Sciences Ahvaz Iran

**Keywords:** BMI1 gene, genetic polymorphisms, leukemia, rs1042059, rs11591377

## Abstract

**Background and Aims:**

Leukemia is a heterogeneous group of hematologic malignancies influenced by both genetic and environmental factors. The B‐cell‐specific Moloney murine leukemia virus integration site 1 (BMI1) gene, a key regulator of hematopoietic stem cell self‐renewal and oncogenesis, has been implicated in leukemia pathogenesis. Among its polymorphisms, rs1042059 and rs11591377 have attracted attention due to their potential association with leukemia susceptibility. This Narrative review aims to explore the roles of these specific single nucleotide polymorphisms (SNPs) in leukemia development.

**Methods:**

We conducted a comprehensive literature review of case‐control studies, genome‐wide association studies (GWAS), and functional analyses evaluating the relationship between BMI1 SNPs (rs1042059 and rs11591377) and leukemia susceptibility.

**Results:**

Some studies have reported significant associations between these polymorphisms and increased leukemia risk, suggesting potential effects on gene expression and leukemogenic signaling pathways. However, results are not consistent across all populations and study designs, indicating the need for further validation.

**Conclusion:**

BMI1 polymorphisms rs1042059 and rs11591377 may contribute to leukemia susceptibility, but current findings remain inconclusive. Future research should focus on clarifying the molecular mechanisms involved and conducting large‐scale, population‐based studies to validate these associations. Understanding these genetic variants may improve leukemia risk assessment and inform personalized therapeutic strategies.

## 1 Introduction

Leukemia is a heterogeneous group of hematologic malignancies characterized by the uncontrolled proliferation of abnormal blood cells. It is classified into several subtypes, including acute lymphoblastic leukemia (ALL), acute myeloid leukemia (AML), chronic lymphocytic leukemia (CLL), and chronic myeloid leukemia (CML), each with distinct genetic and molecular characteristics. While environmental factors such as radiation and chemical exposure contribute to leukemia pathogenesis, genetic predisposition plays a crucial role in disease susceptibility and progression. Identifying genetic variations associated with leukemia risk is essential for understanding its molecular mechanisms and improving diagnostic and therapeutic approaches [1, 2, 3, 4, 5].

The B‐cell‐specific Moloney murine leukemia virus integration site 1 (BMI1) gene encodes a polycomb group (PcG) protein involved in epigenetic regulation, stem cell renewal, and oncogenesis. It plays a vital role in hematopoiesis by maintaining self‐renewal capacity in hematopoietic stem cells (HSCs) and regulating apoptosis and proliferation. Dysregulation of BMI1 has been implicated in various cancers, including leukemia, where its overexpression is often associated with poor prognosis and resistance to therapy. Among the genetic variations within the BMI1 gene, single nucleotide polymorphisms (SNPs) such as rs1042059 and rs11591377 have been suggested to influence leukemia susceptibility by altering gene expression and function [6, 7, 8, 9]. BMI1 was prioritized due to its pivotal role in HSC regulation and leukemogenesis, with focus on rs1042059 and rs11591377 based on recent functional and association evidence, though its overall contribution to leukemia heritability remains modest in polygenic models.

This Narrative review explores the association between rs1042059 and rs11591377 polymorphisms of the BMI1 gene and leukemia risk. We provide an overview of their potential role in leukemogenesis, summarize findings from recent studies, and discuss their clinical significance in risk assessment and therapeutic targeting. Understanding these genetic variations may offer valuable insights into personalized medicine strategies for leukemia management.

## 2 Methods

### 2.1 Literature Search

This Narrative review was conducted as a narrative synthesis to evaluate the potential role of BMI1 gene polymorphisms rs1042059 and rs11591377 in leukemia susceptibility. We aimed to identify studies exploring these SNPs' associations with leukemia risk, functional impacts, and mechanistic roles in leukemogenesis. A targeted literature search was performed using PubMed, Google Scholar, and Web of Science, covering publications up to 2026. Search terms included “BMI1 polymorphism leukemia,” “rs1042059 leukemia,” “rs11591377 ALL/AML/CML,” “BMI1 GWAS leukemia,” and “BMI1 enhancer leukemia.” Reference lists of included studies were manually reviewed to identify additional relevant articles. Although the search was focused rather than exhaustive, the initial yield reflects the specificity of our criteria, which targeted studies directly addressing these SNPs in leukemia contexts. Duplicate and irrelevant records were excluded to ensure all eligible studies were captured within the defined scope.

### 2.2 Eligibility Criteria

Studies were included if they investigated the associations or functional effects of BMI1 SNPs rs1042059 and rs11591377 in leukemia (e.g., AML, ALL, and CML), including case‐control studies, GWAS, fine‐mapping, or preclinical functional analyses. Eligible studies had to report outcomes related to genetic risk, gene expression modulation, or leukemogenic pathways. Only English‐language articles published up to 2026 were considered to ensure accessibility and relevance. Exclusion criteria included studies not specifically addressing these SNPs, research focused on non‐leukemia conditions (e.g., other cancers), and non‐original data sources such as reviews, editorials, or conference abstracts. These criteria ensured that selected studies provided high‐quality insights into the genetic variants' roles in leukemia susceptibility and their potential clinical implications.

### 2.3 Study Selection

Two independent reviewers (M. Aghaei and N. Afrough) assessed the eligibility of the retrieved records according to the pre‐defined inclusion and exclusion criteria. Initially, titles and abstracts were screened for relevance. Subsequently, the full texts of potentially eligible studies were retrieved and evaluated in detail by both reviewers. Any disagreements during the screening or eligibility assessment were resolved through discussion or by consulting a third reviewer (M.A. Jalali Far).

### 2.4 Data Extraction

Data extraction was independently performed by two reviewers using a standardized form. Extracted data included study design, population/ethnicity, sample size, genotype frequencies, *p*‐values, odds ratios, functional impacts (e.g., protein stability and enhancer activity), key findings, and study limitations. This approach minimized bias and ensured comprehensive evaluation of the association between BMI1 SNPs and leukemia susceptibility.

## 3 BMI1 Gene and Its Role in Leukemia

### 3.1 Genomic Localization and Functional Consequences of rs1042059 and rs11591377

The two SNPs selected for this review exhibit distinct genomic positions and functional impacts within or near the BMI1 gene. rs1042059 (G > A) is a nonsynonymous variant located in exon 3 of BMI1 at chromosomal position 10:22326502 (GRCh38) or 10:22615431 (GRCh37). This change results in a C18Y amino acid substitution within the RING finger domain of the BMI1 protein, which is critical for its E3 ubiquitin ligase activity as part of Polycomb Repressive Complex 1 (PRC1). Functional studies indicate that the C18Y substitution increases ubiquitination of BMI1 itself, leading to enhanced proteasomal degradation and consequently reduced BMI1 protein levels and stability [10]. This variant has been directly associated with leukemia susceptibility in a recent case‐control study in an Iranian population, where the GA genotype was significantly more prevalent in patients with AML, ALL, and CML compared to controls (*p* < 0.001) [11].

In contrast, rs11591377 (G > A,T) is situated in an intergenic region upstream of BMI1 at chromosomal position 10:22134373 (GRCh38) or 10:22423302 (GRCh37), within a hematopoietic stem cell‐specific enhancer element. This regulatory variant does not alter the coding sequence but influences BMI1 expression through modulation of transcription factor binding. Fine‐mapping and functional analyses have shown that the risk allele (G) enhances recruitment of MYBL2 and p300 to the enhancer, potentially increasing BMI1 transcription in a cell‐type‐specific manner relevant to hematopoiesis [6]. This SNP has been implicated in childhood ALL through GWAS and fine‐mapping studies, particularly in high‐hyperdiploid subtypes, with consistent associations across multi‐ethnic cohorts [6, 11].

These distinct genomic locations—one coding (rs1042059) and one regulatory (rs11591377)—underlie their potential differential contributions to leukemia risk: reduced protein stability vs. altered expression regulation. Both mechanisms may disrupt normal hematopoietic stem cell homeostasis, promoting leukemogenic transformation. Detailed information is summarized in Table 1.

**Table 1:** Summary of key BMI1 gene polymorphisms and their association with leukemia.

Polymorphism	Chromosome location	Alleles	Associated leukemia type	Functional impact	Ref.
rs1042059	10:22326502 (GRCh38) 10:22615431 (GRCh37)	G > A	AML CML ALL	Resulting in a C18Y amino acid substitution within the RING finger domain of the BMI1 protein, which leads to a significant reduction in BMI1 protein levels due to increased ubiquitination and subsequent proteasomal degradation	[10, 11, 12]
rs11591377	10:22134373 (GRCh38) 10:22423302 (GRCh37)	G > A,T	ALL AML CML	Enhancing MYBL2 and p300 transcription factor binding within a hematopoietic stem cell enhancer, potentially altering BMI1 expression	[6, 11]

### 3.2 Biological Functions of BMI1

BMI1 oncogene is a key part of Polycomb repressor complex 1, involved in many cellular processes through different pathways. It is associated with cell cycle progression, senescence, aging, apoptosis, angiogenesis, and self‐renewal in several lineages of adult stem cells [13]. What makes BMI1 particularly interesting is that many of these functions are the same mechanisms that drive tumorigenesis. Therefore, it is not just that BMI1 happens to be involved in cancer; its fundamental biological roles actively influence the process of tumor development [14]. In addition to its well‐documented contribution to tumorigenesis, BMI1 regulates a wide array of essential biological processes in both malignant and non‐malignant contexts. The key functions of BMI1 are outlined below:

#### 3.2.1 Proliferation

**Figure 1:**
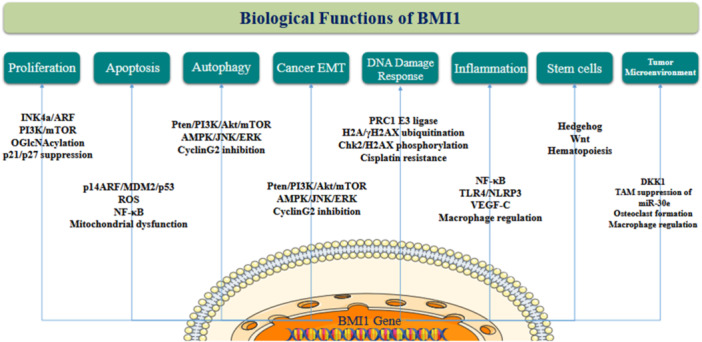
Biological functions of BMI1 and key pathways underlying its functions.

BMI1 plays a pivotal role in cell cycle regulation primarily by repressing the INK4a/ARF locus, which encodes the tumor suppressors p16^INK4a and p14^ARF [15]. p16^INK4a inhibits cell cycle progression at the G0/G1 phase by blocking the cyclin D–CDK4/6 complex, thereby preventing phosphorylation of pRb and subsequent release of E2F transcription factors that drive S‐phase entry. In parallel, p14^ARF stabilizes p53 by inhibiting its interaction with MDM2, thereby promoting p53‐dependent apoptosis and cell cycle arrest in response to oncogenic stress [16, 17, 18, 19]. Through this dual repression of p16^INK4a and p14^ARF, BMI1 promotes cell proliferation and suppresses senescence and apoptosis, mechanisms that are frequently dysregulated in cancer. Conversely, p14^ARF stabilizes p53 by inhibiting its interaction with MDM2, thereby enhancing p53‐mediated transcriptional activation and promoting apoptosis in response to oncogenic or genotoxic stress. In addition, BMI1 directly represses the cyclin‐dependent kinase inhibitors p21^CIP1 and p27^KIP1, thereby relieving inhibition of cyclin‐CDK complexes, facilitating G1/S progression, and promoting cell proliferation [20]. Moreover, by activating the PI3K/mTOR/4EBP1 and O‐GlcNAcylation pathways, BMI1 enhances cell growth in many types of cancers, including colorectal, lung, and ovarian cancers [21, 22, 23, 24, 25, 26]. However, some studies suggest that Bmi‐1 doesn't impact the cell cycle in certain types of cancer, like lung cancer, which may depend on the specific cell type (Figure 1) [27].

#### 3.2.2 Apoptosis

BMI1 affects cell apoptosis via different pathways. The absence of BMI1 causes apoptosis and impairs DNA double‐strand repair through homologous recombination [28, 29, 30]. BMI1 inhibition can lead to p14ARD overexpression and consequently, destabilize p53 through p14ARF/MDM2/p53 and cause apoptosis [28, 31, 32]. Additionally, it can promote ROS‐mediated apoptosis by impairing normal mitochondrial function [33]. Conversely, expression of BMI1 can save cells from apoptosis by activating NF‐κB pathway. Steadily, overexpression of BMI1 is reported to make cells resistant to cisplatin‐induced apoptosis through the same mechanism (Figure 1).

#### 3.2.3 Autophagy

Overexpression of BMI1 boosts both proliferation and migration of cardiac fibroblasts through activating PTEN/PI3K/Akt/mTOR pathway, inhibiting autophagy [34]. It has also been shown to inhibit cyclinG2 and therefore the PKCζ/AMPK/JNK/ERK pathway which is responsible for autophagy [35]. On the other hand, BMI1 knockdown encourages autophagy. It inhibits autophagy in ovarian cancer cells by decreasing ATP. It also influences autophagy in breast cancer [36, 37]. Moreover, sodium butyrate and betulinic acid treatment are reported to reduce BMI1 expression in bladder cancer, leading to autophagy through AMPK/mTOR and BMI‐1/ROS/AMPK/mTOR/ULK1 axis, respectively [17, 33]. Additionally, betulinic acid is also responsible for promoting apoptosis (Figure 1).

#### 3.2.4 Cancer EMT

Expression of BMI1 is predominantly related to promoting migration, invasion, and metastasis through multiple mechanisms in many types of cancer. BMI1 knockdown up‐regulates E‐cadherin and keratin unlike N‐Cadherin, vimentin, and SLUG, decreasing the migration and invasion of endometrial cancer cells both in vivo and in vitro [38]. Moreover, by influencing miR‐27a and miR‐155, which suppress raf kinase inhibitory protein (RKIP), BMI1 increases migration in gastric cancer [39]. It also inhibits PTEN, activates PI3K/Akt pathway and enhances MMP2, MMP9, and VEGF expression [40]. Through this mechanism, BMI1 promotes the migration and invasion of cancer cells in hepatocellular carcinoma, breast cancer, colon cancer, and esophageal cancer [39]. Additionally, BMI1 promotes NF‐κB‐driven MMP9 transcription in glioma and activates the TLR4/MD2/MyD88‐NF‐κB pathway in colorectal cancer cell EMT [41, 42]. Conversely, miR‐218, miR‐330‐3p, and miR‐498 inhibit BMI1 through the BMI1/Akt axis, reducing migration and invasion [42].

#### 3.2.5 DNA Damage Response

BMI1 plays a crucial role in maintaining genomic stability by regulating the DNA damage response (DDR) [43, 44]. Its absence leads to mitochondrial dysfunction and increased ROS, triggering DDR activation [45]. As part of PRC1, BMI1 forms an E3 ubiquitin ligase with RING1B, promoting histone H2A and γH2AX ubiquitination to facilitate DNA repair via homologous recombination and non‐homologous end joining [46, 47]. BMI1 also reduces genotoxic stress from ionizing radiation [48]. Its loss worsens cisplatin‐induced DNA damage, increasing Chk2 and H2AX phosphorylation, underscoring its role in DNA repair and cancer progression [49].

#### 3.2.6 Inflammation

BMI1 has been found as a regulator of NF‐κB transcription factors and is considered as the key component of inflammation. Through this mechanism, it increases proliferation, metastasis, and drug resistance of the tumor cells [50]. It has been seen to activate NF‐κB/MMP3 or NF‐κB/MMP9 signaling in glioma, while regulating TLR4/MD2/MyD88 complex‐mediated NF‐κB signaling pathway in colorectal cancer [41, 51]. Bmi‐1 also supports glioma angiogenesis by boosting VEGF‐C [52]. TLR4, however, inhibits Bmi‐1 and activates the NLRP3 inflammasome pathway to drive inflammation (Figure 1) [53].

#### 3.2.7 Stem Cells

BMI1 is responsible for self‐renewal and differentiation of cancer stem cells. It is essential in maintaining the self‐renewal abilities of natural stem cells [54, 55, 56, 57, 58]. For instance, BMI1 is essential for normal blood cell production, and its absence leads to significant issues [59, 60]. Additionally, Bmi‐1 is regulated by pathways like Hedgehog and Wnt in breast cancer stem cells and hematopoietic stem cells, which help them grow and self‐renew [61]. Bmi‐1 is also involved with prostate and intestinal stem cells and is found in mesenchymal stem cells [62, 63].

#### 3.2.8 Tumor Microenvironment

BMI‐1 expression can influence the TME. For example, the BMI‐1 inhibitor PTC‐209 increases DKK1 expression by down‐regulating BMI‐1, which impairs osteoclast formation and disrupts the TME [64]. BMI‐1 is also upregulated in macrophages associated with multiple myeloma (MM), where it supports their pro‐myeloma functions. Inhibiting BMI‐1 can target both MM cells and macrophages [65]. Additionally, tumor‐associated macrophages (TAMs) may enhance BMI‐1 expression in gastrointestinal cancer by suppressing miR‐30e, promoting cancer progression. Further research is necessary to fully understand BMI‐1's impact on the TME (Figure 1) [66].

## 4 BMI1 Role in Leukemia

Leukemia is driven by a complex network of genetic and epigenetic factors, and BMI1, plays a crucial role in this process. It regulates self‐renewal and differentiation in leukemic stem cells (LSCs), which are implicated in disease relapse and poor prognosis in various leukemia types, including ALL, AML, and CML. Beyond its role in LSC maintenance, variations in the BMI1 gene itself may contribute to leukemia risk. Studies have identified two key polymorphisms, rs1042059 and rs11591377, that appear significantly more frequently in leukemia patients than in healthy individuals [11]. The GA genotype of these variants may influence BMI1 expression, potentially altering the regulation of hematopoietic stem cells and pushing them toward malignant transformation [11]. These findings not only reinforce BMI1's role in leukemia progression but also raise the possibility of using it as a genetic marker for susceptibility and prognosis [11]. See Table 1.

Ohtaka and colleagues were among the first to suggest that inhibiting BMI1 could suppress the growth of leukemia cells by impacting the NOTCH signaling pathway, which acts downstream of BMI1 [67]. This was unexpected because earlier research had pointed to NOTCH signaling as being upstream of BMI1, especially in T‐cells and intestinal stem cells [68, 69]. They investigated the effects of three BMI1 inhibitors—artemisinin, PTC‐209, and PRT4165—on leukemia cell lines, including those from AML and T‐ALL. The results showed that PTC‐209 and PRT4165 effectively suppressed the growth of all leukemia cells tested, triggering apoptosis without affecting normal lymphocytes. These inhibitors not only reduced BMI1 expression but also disrupted key signaling pathways like NOTCH1, HES1, and MYC, which are vital for cell proliferation [67]. Microarray analysis confirmed that the inhibitors lowered the expression of several genes involved in leukemia growth. Additionally, WY332, a small‐molecule inhibitor, effectively reduced Ring1B‐BMI1‐mediated histone modification (H2AK119ub) in leukemia cells, inducing cellular differentiation and altering colony‐forming potential [70, 71]. This inhibition led to reprogramming of gene expression and impaired LSC self‐renewal (Table 2).

**Table 2:** Role of BMI1 in leukemia pathogenesis and therapeutic targeting.

Function of BMI1	Molecular mechanism	Effect on leukemia	Therapeutic targeting	Ref.
Cell proliferation	Regulates NOTCH1, HES1, MYC, and the polycomb repressive complex 1 (PRC1); silences tumor suppressor genes (p16 and p14ARF) via chromatin remodeling.	Promotes leukemia cell proliferation in AML, T‐ALL, childhood ALL, Ph+ ALL, and CALM‐AF10 AML; enhances c‐Myc‐mediated oncogenesis.	Inhibitors like PTC‐209, PRT4165, and WY332 disrupt BMI1‐driven proliferation and NOTCH1 signaling. IFNα therapy downregulates BMI1 in Ph+ ALL, reducing cell growth.	[67, 70, 71, 72]
Apoptosis resistance	Suppresses pro‐apoptotic genes (BIM and E2F7) and interacts with LncRNA LINC01255 to repress MCP‐1 transcription, thereby activating p53‐p21 and preventing senescence.	Contributes to chemoresistance and leukemia progression in AML and Ph+ ALL; promotes survival in leukemic cells by evading apoptosis.	RNA interference silencing BMI1 increases BIM and E2F7, restoring apoptotic pathways. siRNA nanocarriers (PEI@HSANCs) trigger caspase‐3‐mediated apoptosis.	[72, 73, 74]
DNA damage response	Histone modification (H2AK119ub) via the Ring1B‐BMI1 complex prevents DNA damage‐induced apoptosis; maintains genomic integrity in leukemic cells.	Enables LSC survival and genomic instability, fostering leukemic progression in AML, CML, and T‐ALL.	WY332 disrupts H2AK119ub, promoting differentiation and impairing LSC self‐renewal. BMI1 inhibitors reprogram epigenetic regulation, reversing leukemic stemness.	[70, 71, 74]
Leukemic stem cell maintenance	Essential for LSC self‐renewal; BMI1 is co‐expressed with CD26+ in CML LSCs, allowing for treatment evasion and disease persistence.	BMI1 overexpression correlates with worse survival, relapse, and treatment resistance in AML, CML, MM, and follicular lymphoma.	siRNA nanocarriers (PEI@HSANCs) selectively target BMI1, reducing LSC burden and enhancing differentiation (CD11b+ myeloid shift). BMI1 inhibitors impair therapy‐resistant LSCs.	[73, 74, 75, 76, 77, 78]

A recent study on CML patients identified the co‐expression of BMI1 with CD26+ in leukemia stem cells, supporting previous findings that BMI1 holds diagnostic value in CML [75, 76]. BMI1 expression was significantly higher in the blastic and accelerated phases compared to the chronic phase [75, 76, 77]. Moreover, elevated BMI1 levels in AML patients were associated with worse overall and disease‐free survival, emphasizing its prognostic significance [75, 76, 77, 78].

LncRNA LINC01255 is highly expressed in AML and linked to poor survival. It interacts with BMI1 to repress MCP‐1 transcription, activating the p53–p21 pathway. This prevents senescence in mesenchymal stromal cells and supports AML cell proliferation (Table 2) [73].

As BMI1 is linked to poor prognosis in AML, and targeting it with siRNA nanocarriers (PEI@HSANCs) offers a potential therapy [74]. These nanocarriers enhance BMI1 siRNA delivery, leading to Bax activation, caspase 3‐mediated apoptosis, and suppression of polycomb proteins (BMI1 and EZH2). The treatment downregulates BMI1 via ubiquitin‐mediated degradation, which is reversed by proteasome inhibition. C‐Myb directly regulates BMI1, as shown by ChIP assays. In an AML xenograft model, this approach reduced leukemic stem cells (CD45 +) and promoted myeloid differentiation (CD11b +), highlighting its potential as an epigenetic‐based antileukemic therapy (Table 2) [74].

This correlation between BMI1 expression and disease severity isn't just limited to leukemia; in Primary myelofibrosis (PMF), loss of BMI1 in animal models causes overexpression of the oncogene Hmga2, triggering myeloproliferative conditions and abnormal blood cell production [79]. In multiple myeloma (MM), BMI1 is also overexpressed, and this is linked to disease progression, relapse, and poor outcomes [80]. Additionally, Quantifying BMI1 in B‐cells derived from follicular lymphoma patients has shown that abundant BMI is correlated with poor prognosis [81].

Similarly, in childhood ALL, BMI1 contributes to cell proliferation and survival. Fine‐mapping of chromosome 10p12.31 identified rs11591377, a variant in an enhancer element that may increase BMI1 expression. As part of the polycomb repressive complex 1, BMI1 suppresses p16 and p14ARF, key regulators of the cell cycle encoded by CDKN2A, a frequently deleted gene in ALL. Overexpression of BMI1, influenced by c‐Myc, extends cell lifespan and reduces apoptosis in hematopoietic stem cells. Additionally, BMI1 impacts blood cell development, with genetic studies linking it to increased myeloid cell counts, highlighting its role in both leukemia progression and hematopoiesis [6].

In Ph+ ALL, BMI1 is essential for cell proliferation, survival, and leukemogenesis. Silencing BMI1 upregulates BIM, IFNα‐regulated genes, and E2F7, thereby triggering apoptosis and reducing proliferation. Rescue experiments confirmed that BMI1's oncogenic effects are mediated through the suppression of BIM and E2F7. Furthermore, IFNα treatment was found to inhibit Ph+ ALL cell growth, highlighting BMI1 as a promising therapeutic target in Ph+ ALL (Table 2) [72].

In CALM‐AF10‐rearranged leukemias, BMI1 is significantly upregulated, likely due to transcriptional activation by the fusion protein or disruption of topologically associated domains (TADs). Studies indicate that BMI1 dependency varies across AML subtypes. While AML driven by AML1‐ETO and PLZF‐RARA is highly sensitive to BMI1 depletion, MLL‐AF9‐driven leukemia shows limited dependence. However, CALM‐AF10‐driven leukemias appear to rely on BMI1 for both initiation and maintenance, making BMI1 inhibition a promising therapeutic approach. Preclinical studies demonstrate that a broad range of AML cell lines respond to BMI1 inhibitors, supporting their potential clinical use, particularly in CALM‐AF10‐associated AML. Given that BMI1 overexpression is also observed in T‐ALL, future studies will focus on developing CALM‐AF10 T‐ALL models to further validate the efficacy of BMI1 inhibitors in these leukemias [82].

These findings suggest that BMI1 is not only a regulator of LSCs but also a potential marker for leukemia risk and progression. Overlooking BMI1 in treatment strategies and failing to assess its role in different individuals could be the missing link in understanding relapse and resistance. Additionally, studying its polymorphisms could lead to better risk assessment and more effective targeted therapies. In prior studies, rs11591377 (risk allele G, more prevalent in European‐ancestry and admixed populations with RAF ~ 0.77–0.91 and OR 1.27 for childhood ALL) has shown consistent associations via enhancer modulation [6], while rs1042059 (GA genotype enrichment) is prominently reported in Iranian cohorts [11], highlighting potential population‐specific effects.

## 5 Limitations and Future Perspectives

Despite growing evidence on the role of BMI1 gene polymorphisms in leukemia susceptibility, several limitations exist. Many studies suffer from small sample sizes and lack replication across diverse populations, necessitating larger, multi‐ethnic, and well‐powered studies to confirm these associations. The variability in polymorphism frequency across different ethnic groups further emphasizes the need for comprehensive genome‐wide investigations. Additionally, while statistical correlations between rs1042059 and rs11591377 and leukemia have been reported, functional validation remains limited. Future studies should employ gene‐editing techniques such as CRISPR/Cas9 to determine their precise effects on BMI1 expression and function. Another important gap is the limited understanding of how these polymorphisms interact with environmental and epigenetic factors, which may play a crucial role in leukemia risk. Moreover, translating these findings into clinical practice requires extensive validation through prospective studies and meta‐analyses. Investigating the impact of these polymorphisms on leukemia progression, treatment response, and resistance mechanisms could offer valuable insights for precision medicine. Furthermore, integrating BMI1 polymorphisms with other leukemia‐associated genetic markers may improve risk prediction models, ultimately enhancing early detection and personalized therapeutic strategies.

## 6 Conclusion

The BMI1 gene plays a crucial role in hematopoietic stem cell regulation and leukemogenesis. While rs1042059 and rs11591377 polymorphisms have been linked to leukemia susceptibility, inconsistencies across studies indicate the need for further research. Future large‐scale and functional studies are essential to confirm their clinical significance and uncover their mechanistic roles. Understanding these genetic variations will not only enhance leukemia risk assessment but also contribute to the development of targeted therapeutic interventions, advancing personalized medicine in leukemia management.

## Author Contributions

**Mojtaba Aghaei:** writing – original draft, writing – review and editing, data curation, conceptualization, validation, project administration. **Niloufar Afrough:** writing – original draft, writing – review and editing, data curation, visualization. **Mohammad Ali Jalali Far:** supervision, conceptualization, validation, writing – review and editing, project administration.

## Funding

The authors have nothing to report.

## Ethics Statement

The authors have nothing to report.

## Consent

The authors have nothing to report.

## Conflicts of Interest

The authors declare no conflicts of interest.

## Transparency Statement

The lead author Mohammad Ali Jalali Far affirms that this manuscript is an honest, accurate, and transparent account of the study being reported; that no important aspects of the study have been omitted; and that any discrepancies from the study as planned (and, if relevant, registered) have been explained.

## Data Availability

Data sharing is not applicable to this article, as no new data were created or analyzed in this study.
